# A Case of Pseudohyperaldosteronism Induced by Yokukansan and Shakuyakukanzoto That Resulted in Severe Hypokalemia

**DOI:** 10.7759/cureus.38267

**Published:** 2023-04-28

**Authors:** Mika Arai, Hiroki Isono, Momoko Isono, Kousuke Ihara, Keisuke Kondo

**Affiliations:** 1 Department of General Medicine, HITO Medical Center, Ehime, JPN

**Keywords:** kampo medicines, potassium, licorice, pseudohyperaldosteronism, hypokalemia

## Abstract

Pseudohyperaldosteronism can be induced by the excessive use of Chinese herbal medicines (Kampo medicines), resulting in serious disorders. We report a case of pseudohyperaldosteronism induced by two Kampo medicines which resulted in severe hypokalemia. A 70-year-old woman was hospitalized for a left calcaneal fracture. She had no subjective symptoms other than trauma. On her admission, blood test results revealed a low serum potassium level of 2.4 mmol/L by chance, as well as low levels of both renin and aldosterone. The patient had been taking 5 g of Yokukansan per day for the past three months. In addition, she was on 5 g Shakuyakukanzoto per day for three months until a month prior to hospitalization. The daily licorice content from the aforementioned herbs was 1.0 g and 4.0 g, respectively. After hospitalization, the administration of the Kampo medicines was discontinued, and 610 mmol of potassium was administered over a period of 13 days, which resulted in the normalization of serum potassium levels. Pre-existing hypertension slowly improved as well. Patients consuming licorice at doses of 2.5 g per day or more, as in our case, are at a high risk of developing pseudohyperaldosteronism. Furthermore, the risk is particularly high with long-term licorice consumption as well as for women and elderly patients. To this end, high-dose potassium supplementation may be necessary for normalizing serum potassium levels. Therefore, awareness regarding the adverse effects of licorice is crucial, even in cases of low dosages of licorice.

## Introduction

Pseudohyperaldosteronism presents with symptoms of hyperaldosteronism, such as hypertension, edema, and low blood potassium levels without high aldosterone levels. It may be induced by the ingestion of Kampo medicines that contain licorice and occasionally results in severe clinical illness. There have been more than 200 recorded cases of licorice-induced pseudohyperaldosteronism [1,2]. However, reports mentioning potassium supplementation are rare. In this study, we report a case of pseudohyperaldosteronism induced by Chinese traditional medicines Yokukansan and Shakuyakukanzoto that resulted in severe hypokalemia.

Yokukansan (Tsumura TJ-54) has been approved in Japan to treat patients with nervousness and insomnia, as well as night terrors and temper tantrums in children [3]. Shakuyakukanzoto has been prescribed in Japan as an antispasmodic drug for the treatment of skeletal muscle cramps and intestinal cramps [4]. As both these medicines are not only prescription drugs but are also available at pharmacies, awareness regarding their serious side effects is needed.

## Case presentation

A 70-year-old Japanese woman was injured while climbing over a 1-m-high window frame. After visiting another hospital, she was diagnosed with a left heel (calcaneal) bone fracture and was referred to our orthopedic department for admission. On admission, her blood test results showed hypokalemia; thus, she was transferred to the General Medicine Department for consultation. A review of systems was negative except for heel pain.

The patient’s medical history included depression and hypertension for the last two months. Hypertension was pointed out by the doctor during a blood pressure check at a regular clinical visit. She had been taking 5 g of Yokukansan per day for the last three months and amlodipine 7.5 g for the last two months. In addition, she had been on 5 g of Shakuyakukanzoto per day for three months until a month prior to hospitalization. Her daily doses of licorice from the above-mentioned Kampo medicines were 1.0 g and 4.0 g, respectively. The patient’s height, weight, and body mass index were 152.5 cm, 43.5 kg, and 18.7 kg/m2, respectively. On admission, the patient was clearly conscious, and her vital signs were as follows: body temperature 36.8 ℃, pulse rate 74 bpm regular, and blood pressure 160/94 mmHg. Other than the immobilization of the left lower extremity in a cast, there were no abnormal findings on physical examination.

Biochemical testing showed high sodium and low potassium and chloride level (Table 1). Blood gas analysis collected in room air showed the existence of metabolic alkalosis with high bicarbonate levels. Her EKG revealed QT prolongation and a pulse rate of 73 per minute with sinus rhythm. Rate-corrected QT interval (QTc) was 530 ms.

**Table 1:** Laboratory tests WBC: white blood cell, RBC: red blood cell, Hb: hemoglobin, Plt: platelet, Neut: neutrophils, LD (IFCC): lactate dehydrogenase, Alb: albumin, BUN: blood urea nitrogen, Cr: creatinine, Na: sodium, K: potassium, Cl: chlorine, Mg: magnesium, Ca: calcium, CK: creatine kinase pH: potential hydrogen, Pco2: partial pressure of carbon dioxyde, Po2: partial pressure of oxygen, Hco3: bicarbonate, O2 SAT: oxygen saturation, AnGap: anion gap U-Na: urine sodium, U-K: urine potassium, U-Cl: urine chlorine, U-Cr: urine creatinine

Laboratory tests	On the day of presentation	Reference range
WBC	4800/μL	3300-8600/μL
RBC	3.97 million/μL	3.86-4.92 million/μL
Hb	11.8 g/dL	11.6-14.8 g/dL
Plt	200,000/μL	158,000-348,000/μL
Neut.	75%	37-74%
LD (IFCC)	462 U/L	124-222 U/L
Alb	3.0 g/dL	4.1-5.1 g/dL
BUN	14.2 mg/dL	8.0-20.0 mg/dL
Cr	0.67 mg/dL	0.46-0.79 mg/dL
Na	146 mmol/L	138-145 mmol/L
K	2.4 mmol/L	3.6-4.8 mmol/L
Cl	94 mmol/L	101-108 mmol/L
Mg	2.1 mmol/dL	1.8-2.4 mmol/L
Ca	8.9 mg/dL	8.8-10.1 mg/dL
CK	633 U/L	41-153 U/L
Blood gas		
pH	7.557	7.350-7.450
Pco2	49.7 mmHg	35.0-45.0 mmHg
Po2	61.8 mmHg	80.0-100.0 mmHg
Hco3	43.2 mEq/L	22.0-26.0 mEq/L
O2 SAT	94.0%	94.6-98.2 %
NA+	143.0 mmol/L	-
K+	2.5 mmol/L	-
Cl	98.0 mmol/L	-
AnGap	4.7 mmol/L	10.0-18.0
Urinalysis results		
pH	8.0	-
U-Na	128 mmol/L	-
U-K	24.5 mmol/L	-
U-Cl	103 mmol/L	-
U-Cr	59.05 mg/dL	-
U-K/U-Cre	0.41	-

**Figure 1:**
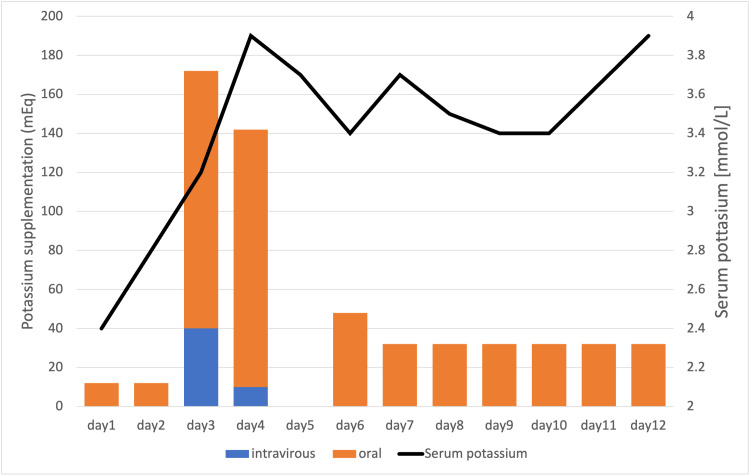
Amount of potassium replacement and serum potassium levels

Licorice-induced pseudohyperaldosteronism was suspected on the basis of the patient's medication history. On days 1 and 2 of hospitalization, the patient was managed in the orthopedic department and was given only small amounts of potassium supplementation. Because of the severe hypokalemia, she was hospitalized in the high-care unit. Yokukansan was discontinued, and an arterial line was inserted to perform blood tests every four to six hours on the third day of hospitalization when the General Medicine Department was involved. Oral potassium chloride was initiated at 160 mmol per day with frequent monitoring on day 3 of hospitalization. In total, 610 mmol (610 mEq) of potassium was supplemented over 13 days, resulting in an increase in her serum potassium concentration to a safe level (Figure 1). Nine days after hospitalization, surgery was conducted on the patient’s heel.

In a blood sample submitted on the third day of hospitalization, both renin and aldosterone exhibited low levels, measuring 0.4 ng/mL/h and ˂4.0 pg/mL, respectively. On the other hand, adrenocorticotropic hormone and cortisol levels were negative for adrenal insufficiency. The patient’s high blood pressure slowly improved after the discontinuation of Yokukansan, and amlodipine was discontinued. Moreover, her serum potassium concentration remained normal. Therefore, her diagnosis of licorice-induced pseudohyperaldosteronism was confirmed.

## Discussion

In this case, we encountered a patient with hypokalemia caused by licorice-induced pseudohyperaldosteronism. Although their name does not indicate so, some Kampo medicines contain licorice. Therefore, it is important to be aware of the side effects (such as pseudohyperaldosteronism) that could arise from using such Kampo products. This will allow necessary measures to be taken to reverse the side effects. In this case, the severe hypokalemia caused by pseudohyperaldosteronism was treated with substantial potassium supplementation. Other major side effects resulting from the use of Kampo medicines are interstitial pneumonia and liver dysfunction.

It has been reported that the risk of licorice-induced pseudohyperaldosteronism is usually high when the daily intake of licorice is more than 2.5 g, especially when the patient is elderly, female, or has been consuming licorice in the long term [5]. In this case, the patient was an elderly female of small stature, who was administered 5.0 g Kampo medicines per day for more than three months. Therefore, she was at a high risk of developing pseudohyperaldosteronism. However, the two types of Kampo medicines she was taking were prescribed by two different clinics, neither of which monitored her potassium levels.

A previous report found that for every 100 mEq decrease in systemic potassium stores, serum potassium concentration decreased by approximately 0.27 mEq/L [6], and in chronic hypokalemia, a potassium deficiency of 200-400 mEq/L decreased serum potassium concentration by 1 mEq/L [7]. In the current case, the low serum potassium level (2.4 mmol/L) of our patient on admission was normalized after supplementation with 610 mmol of potassium, which is similar to the quantities reported in the above-mentioned studies [6,7]. A Japanese case study reported that an aldosterone antagonist may be useful in reducing the dose needed for potassium replacement [8]. However, we chose not to administer this medication as it takes some time to attain therapeutic hormone levels. Additionally, the use of aldosterone antagonists for pseudohyperaldosteronism is not covered by insurance in Japan, and ethical approval is required for the administration of drugs for reasons not covered by insurance. Furthermore, there are no standardized dosing guidelines for aldosterone antagonists.

Licorice-induced pseudohyperaldosteronism can sometimes result in severe side effects such as hypertensive crisis [9], recurrent ventricular fibrillation [10], syncope [11], and torsade de pointes [12]. This patient could have developed any of these conditions if her hypokalemia was not identified by chance upon presenting with a left heel bone fracture.

## Conclusions

For the management of hypokalemia caused by licorice-induced pseudohyperaldosteronism, a substantial amount of potassium supplementation is required. Licorice-induced pseudohyperaldosteronism may result in clinically severe conditions. Owing to this, vigilance is required in identifying side effects associated with licorice administration, even in small doses. This is especially important in elderly and female patients as well as those on prolonged therapy.
